# Comparative analyses of chloroplast genomes of *Theobroma cacao* from northern Peru

**DOI:** 10.1371/journal.pone.0316148

**Published:** 2025-03-05

**Authors:** Daniel Tineo, Danilo E. Bustamante, Martha S. Calderon, Manuel Oliva

**Affiliations:** 1 Instituto de Investigación para el Desarrollo Sustentable de Ceja de Selva (INDES–CES), Universidad Nacional Toribio Rodríguez de Mendoza, Chachapoyas, Amazonas, Perú; 2 Instituto de Investigación de Ingeniería Ambiental, Facultad de Ingeniería Civil y Ambiental (FICIAM), Universidad Nacional Toribio Rodríguez de Mendoza, Chachapoyas, Amazonas, Perú; USDA-ARS Southeast Area, UNITED STATES OF AMERICA

## Abstract

*Theobroma cacao* is the most economically important species within the genus *Theobroma*. Despite its importance, the intraspecific relationships of this species has not been fully elucidated due to insufficient molecular information. To facilitate a better understanding of the intraspecific evolutionary relationships of *T. cacao*, Sequencing technology has been to decode the plastid genomes, with the objective of identify potential DNA barcode genetic markers, explore intraspecific relationships, and infer divergence times. The plastid genome of the seven cocoa genotypes analyzed in this study, exhibited a typical angiosperm genomic structure. However, the structure of each plastid genome reflects notable changes in each genotype; for example, the *infA* gene was present in all the analyzed samples, unlike in previously published cocoa plastid genomes, while the complete *ycf1* gene sequence has potential for use as DNA Barcoding in *T. cacao*. The estimated age of the node connecting *T. cacao* and *T. grandiflorum*, which was 10.11 Ma, supports this indication. It can be inferred that *T. cacao* diverged at approximately 7.55 Ma, and it is highly likely that *T. cacao* populations diversified during the Pliocene or Miocene. Therefore, it is crucial to perform mitochondrial and nuclear-based analyses on a broader spectrum of cocoa samples to validate these evolutionary mechanisms, including genetic estimates and divergence. This approach enables a deeper understanding of the evolutionary relationships among cocoa.

## Introduction

*Theobroma* L. is a genus within the Malvaceae family that encompasses 22 species [1]. The most economically important species is *Theobroma cacao* L. [2]. This tropical understory tree originated in the Amazon basin in South America but grown as a commercial crop on plantations in Africa, Asia, and America, as it is an important source of income for many farmers in those regions [3,4]. According to Utro et al. [5], cocoa is among the ten principal agricultural commodities worldwide. The high market value of *T. cacao* is attributed to the flavonoids it contains. These secondary metabolites are associated with numerous health benefits, such as reducing the risk of cardiovascular diseases [6]. Apart from being the source of chocolate, cocoa beans offer carbohydrates, fats, proteins, natural minerals, and vitamins [7].

The cocoa industry traditionally distinguishes three main types of cocoa: Forastero, Criollo, and Trinitario. These cocoa varieties are naturally distributed from southern Bolivia to Mexico [8–11]. While there is some historical ambiguity surrounding their nomenclature, the Forastero variety is commonly recognized in the industry for its sturdiness and features dark purple kernels, which have a bitter taste and sometimes a sour flavor [10,12]. The Criollo variety is less resilient than the other varieties, resulting in the production of kernels that are lightly pigmented or white. These kernels possess a desirable aroma and slight bitterness [13,14]. On the other hand, Trinitario cultivars exhibit high yield and disease resistance, producing kernels that have a much milder taste [10,15,16]. It has been suggested Trinitario may be a hybrid resulting from a combination of Forastero and Criollo varieties [17–19]. Recent scientific research utilizing microsatellite markers has identified ten distinct genetic clusters of cacao [20]. These groups are distributed throughout various South American countries. Amelonado is found in Brazil, Costa Rica, and Ghana; Contamana and Iquitos can be found in Peru and Brazil; Criollo is present in Ecuador, Venezuela, Panama, Costa Rica, and Mexico; National and Curaray are exclusive to Ecuador. Guiana is solely present in Brazil, while Marañón can be found in Peru and Bolivia. Nanay is exclusive to Peru, while Purús is found in Brazil and Bolivia [8]. Moreover, recent studies conducted by Zhang et al. [20], Motamayor et al. [11], and Osorio-Guarín et al. [21] indicated the presence of further cacao populations in Bolivia, Peru, and Colombia, respectively. It is probable that further distinguishable genetic clusters of cocoa will emerge with the increase in exploration of the untamed territories of South America [22]. However, Single Nucleotide Polymorphism (SNP) analyses is another valuable method to elucidate genetic diversity and identify variations in plant genomes [20]. Despite this potential, high-yield genotyping is economically unfeasible in several developing countries where cacao cultivation occurs [4]. These polymorphic DNA sequences and regions may be valuable for evolutionary and phylogenetic research on the genus *Theobroma* and family Malvaceae in the future. This approach allows us to elucidate taxonomic ambiguities and pinpoint taxa closely associated with cocoa farming. The designed markers might also prove useful in distinguishing between genetically similar cultivars and wild taxa for breeding initiatives [22]. Recent updates of the *T. cacao* genome have provided a new and approachable structure for exploring evolutionary proceedings, structural and functional genetics, biochemistry, and comparative genomics of the cacao tree [13,14].

The Amazonas region in Peru is the seventh most productive region for Fine Aroma Cocoa, which is renowned for its distinctive aroma and taste and holds great esteem in the global marketplace [23]. In 2015, the Regional Government of Amazonas introduced the denomination of origin for “Cacao Amazonas Peru” via the Regional Ordinance N° 368 [24]. The decision was made based on cocoa’s bromatological qualities, its growing environment, and the Amazonas region’s important role in its genetic diversity [23,24]. In this region, numerous studies have investigated volatile fingerprints [25]; fatty acids [26]; phenolic, aromatic and physicochemical compounds [27,28]; and phenotypic traits [29,30]. However, a detailed molecular characterization of Fine Aroma Cocoa is still lacking [28]. The only genetic study that supports the genetic diversity of fine aroma cocoa in this region is described by Bustamante el al [31], who using genotyping technology reported the presence of ten genetic groups described by Motamayor et al. [11]. However, the genome structure and functionality of Amazonas Fine Aroma Cocoa have not been fully elucidated, since a structural and functional characterization will allow determining the expression and function of genes associated with various traits in cocoa, such as genes associated with various biological interactions between the cacao tree and diseases such as *Phytophthora* [13,32]. Therefore, the aforementioned methods are crucial for expediting the advancement of potential cultivars via the utilization of pioneering biotechnological methods [33].

In this study, we sequenced and assembled nine complete plastid genomes sourced from Fine Aroma Cocoa (*T. cacao*) cultivation in northeastern Peru. We scrutinized each genome to identify potential DNA barcode genetic markers, explore intraspecific relationships, and infer divergence times when compared to other available plastid genomes in the GenBank database. These findings may assist in distinguishing distinct Fine Aroma Cocoa varieties.

## Materials and methods

### Fine aroma cocoa sample collection

Fine Aroma Cocoa samples were collected in Bagua and Utcubamba provinces (665–902 m.a.s.l.) of the Amazonas region in northeastern Peru (Table 1). Servicio Nacional Forestal de Fauna y Flora Silvestre (SERFOR) granted a wild flora scientific research permit for the collection of Fine Aroma Cocoa (MIDAGRI-SERFOR-DGGSPFFS, with authorization code N° AUT-IFL-2020-0051). Samples were obtained from seven Fine Aroma Cocoa genotypes as described by Bustamante et al. [31] in the Bagua and Utcubamba provinces of the Amazonas region (S1 Fig). Approximately 100 mm^2^ of tender cacao leaves were collected for molecular analyses and placed in pre-labeled 2 mL Eppendorf Safelock tubes. The aforementioned samples were deposited in the KUELAP herbarium under the National University Toribio Rodríguez de Mendoza [34]. The deposit includes comprehensive information pertaining to the sampling sites alongside the characteristics of the plants sampled. Information such as the collection code, date, altitude, locality and GPS coordinates was recorded for each collection site. The voucher codes for each sample are: KUELAP-611, KUELAP-619, KUELAP-638, KUELAP-646, KUELAP-655, KUELAP-659 and KUELAP-663 (Table 1).

**Table 1:** Collection codes for samples of fine aroma cocoa (*T. cacao*).

N°	Sample code	Voucher code	Collection date	Region	Province	Place	Altitude	UTM	Coordinates	
1	INDES6	KUELAP-611	15/01/2018	Amazonas	Utcubamba	El Chalan	754	17	787,894	9,369,168
2	INDES14	KUELAP-619	15/02/2018	Amazonas	Utcubamba	El Limoncito	817	17	793,728	9,366,961
3	INDES34	KUELAP-638	23/02/2018	Amazonas	Bagua	Lluhuana	902	17	787,570	9,371,144
4	INDES50	KUELAP-646	23/02/2018	Amazonas	Utcubamba	Diamante Bajo	730	17	794,447	9,366,031
5	INDES63	KUELAP-655	25/02/2018	Amazonas	Utcubamba	Naranjos Altos	727	17	793,806	9,365,734
6	INDES67	KUELAP-659	25/02/2018	Amazonas	Utcubamba	Naranjos Altos	665	17	792,347	9,364,233
7	INDES71	KUELAP-663	3/03/2018	Amazonas	Utcubamba	La Cruz	810	17	786,904	9,370,301

### DNA extraction, sequencing, assembly and annotation

The National University Toribio Rodríguez de Mendoza de Amazonas Laboratory of Molecular Biology and Genomics conducted the DNA extraction. Genomic DNA was obtained using the NucleoSpin Kit (Macherey-Nagel, Düren, Germany). Subsequently, a NanoDrop and Qubit (Thermo Fisher Scientific, Waltham, MA, USA) were used for optical density measurements of the DNA. Genomic DNA was sequenced commercially by Macrogen (Seoul, SouthKorea). Briefly, the concentration and purity of the DNA were verified before library preparation through agarose gel electrophoresis and Agilent Tapestation. The genomic DNA was fragmented and ligated with individual adapters using the Swift 2S Turbo DNA library preparation using PCR with kit from Swift Bioscience, headquartered in Ann Harbor, MI, USA. Next, we evaluated the size distribution and concentration of the resulting library using Qubit and TapeStation. Library sequencing was carried out on the NextSeq 500 platform developed by Illumina, San Diego, CA, in compliance with established procedures. Has been generated paired 150 nucleotide (nt) reads and checked them for data quality using FastQC from the Babraham Institute located in Cambridge, UK. The plastid genomes were assembled using de novo assembly with MEGAHIT [35], SPAdes-3.13.0 software [36], getorganelle v 1.7.5.3 [37] and visualized with Bandage v 0.8.1 [38]. The reference genome employed during the assembly process was *T. cacao* (HQ336404; Jansen et al. [39]). The precision and circularity of the genome were validated by mapping the reads and contigs with the same mapping tool used for reference in Geneious Prime, v. 2020.0.3. The entire chloroplast genome was annotated through MFannot [40], NCBI ORFfinder, and tRNAscan-SE 2.0 [41]. Afterward, comparison with the reference genome in Geneious Prime allowed manual correction.

### Simple sequence repeats and dispersed repeats

The software tool used for identifying SSRs in *T. cacao* genome sequences was the MicroSatellite Identification Program [42], which is accessible via https://pgrc.ipk–gatersleben.de/misa/. The tool employs a range of parameter settings depending on the unit size (nucleotides) of the SSR, varying from 1_10 for mononucleotide repeats to 6_3 for hexanucleotide repeats, using configuration for Malvales described by Beier et al [42]. A minimum separation of 100 base pairs was considered for identifying two SSRs. To compare the genome structures of *T. cacao*, we utilized the Online IRscope program (https://irscope.shinyapps.io/irapp/). This program facilitated a comparison of the positions of the IR, SSC and LSC regions across the 19 cp genomes of *T. cacao*.

### *T. cacao* polymorphism analysis

All *Theobroma* plastid genomes were aligned with MAFFT v. 7.0.17 [43]. Geneious Prime v. 2023.0.3 was used to calculate the number of mutation and indels events employing the approximate p-value calculation method; indels were considered to be events rather than sites in the alignment with a minimum coverage of 1, minimum variation frequency of 0.25, and minimum string bias of p-value = 10^−7^ [44].

### Phylogenomic analysis and search for specific genes

The seven complete plastid genome sequences generated in this study were combined with 13 *Theobroma* plastome sequences obtained from GenBank (Table 2). *Theobroma grandiflorum* (JQ228388, Kane et al. [45]) was used as the outgroup. Sequence alignment was performed with the MAFFT plugin version 7.0.17 [43], while PartitionFinder-2.1.1 [46] was used to select the best suited model for the complete plastid genomes. Phylogenetic trees were created using the maximum likelihood and Bayesian inference methods with IQ-TREE v.2.2.0 software [47]. The test model (-m TEST) [48] was used in conjunction with 1,500 ultrafast bootstrap replicates. To construct the gene tree and intergenic polymorphic regions, gene splitting was performed using the Ape 5.0 package [49] through RStudio statistical software [50]. From this process, bootstrap and UFOBORT files were constructed utilizing 1,500 ultrafast repeats in IQ-TREE v.2.2.0. All the trees produced were combined and analyzed with ASTRAL–III software [51] to establish a consensus tree with 1,500 replicates. The phylogenetic trees were visualized using TreeDyn 198.3 on Phylogeny.fr [52].

**Table 2:** List of sequences of the chloroplast genome of *T. cacao* generated in this study and downloaded from NCBI used for data analysis.

Species	GenBank	Accession	Country	Traditional variety classification	Reference
***T. cacao***	**OP354232**	**INDES06**	**Peru**		**This study**
***T. cacao***	**OP354233**	**INDES14**	**Peru**		**This study**
***T. cacao***	**MZ725364**	**INDES34**	**Peru**		**This study**
***T. cacao***	**OP354234**	**INDES50**	**Peru**		**This study**
***T. cacao***	**OP354235**	**INDES63**	**Peru**		**This study**
***T. cacao***	**MZ725365**	**INDES67**	**Peru**		**This study**
***T. cacao***	**OP354236**	**INDES71**	**Peru**		**This study**
*T. cacao*	JQ228387	TARS 16664	Trinidad and Tobago	Trinitario	Kane et al. 2012
*T. cacao*	JQ228386	TARS 12044	Trinidad and Tobago	Trinitario (Criollo-type)	Kane et al. 2012
*T. cacao*	JQ228385	MIA 27956	Suriname	Trinitario with similarities to lower Amazon Forastero	Kane et al. 2012
*T. cacao*	JQ228381	TARS 16664	Trinidad and Tobago	Trinitario	Kane et al. 2012
*T. cacao*	JQ228383	TARS 16658	Trinidad and Tobago	Trinitario	Kane et al. 2012
*T. cacao*	KY085907	–	–	–	unpublished
*T. cacao*	JQ228380	TARS 16542	Ghana	Lower Upper Amazon Forastero	Kane et al. 2012
*T. cacao*	JQ228389	PI 275669	–	Lower Upper Amazon Forastero	Kane et al. 2012
*T. cacao*	HQ336404	–	–	Lower Upper Amazon Forastero	Jansen et al, 2010
*T. cacao*	HQ244500	–	Peru	Upper Amazon Forastero, Peru	Kane et al. 2012
*T. cacao*	JQ228382	MIA 29885	Peru	Upper Amazon Forastero, Peru	Kane et al. 2012
*T. cacao*	JQ228379	Criollo-22	Trinidad and Tobago	Pure Criollo variety	Kane et al. 2012
*T. grandiflorum*	JQ228388	04-0254	Puerto Rico	Species related to *T. cacao*. Wild and cultivated in Amazon Basin	Kane et al. 2012

### Estimation of *T. cacao* divergence time

The estimation of the divergence time of Malvaceae was initially conducted using 38 plastome sequences obtained from GenBank (S1 Table). The outgroup consisted of *Carica papaya* (EU431223), *Mangifera indica* (KX871231), and *Tapiscia sinensis* (MF926267). All CDSs found in each species were extracted manually through the use of Geneious Prime, v. 2023.0.3. The CDS dataset was analyzed on the CIPRES Science Gateway portal using a xml input file produced in BEAUti v.1.7.2 [53] within BEAST v1.10.4 [53]. The superior evolutionary model was determined according to the results of PartitionFinder-2.1.1 (GTR +  I +  G substitution model). BEAST analyses were conducted using an a priori birth-death speciation model [54] and an uncorrelated relaxed clock model [55] with a lognormal distribution. To constrain the age of the crown node of Malvaceae, fossil-based calibration points were employed and set to 70.7 Ma with a normal prior and standard deviation equal to 5, following the work of Wang et al. [56]. Four BEAST runs were executed for 400,000,000 generations each, with parameters sampled every 1.000 generations. The effective sample size (ESS > 200) was determined using Tracer v1.7 [57], with 25% of the samples removed as burn-in and 30% of the trees discarded. We employed TreeAnnotator v1.8.4 [58] to generate the maximum clade credibility (MCC) tree displaying mean divergence time estimates alongside 95% highest posterior density (HPD) intervals. Based on these findings, divergence was exclusively carried out for the *Theobroma* genus using the same parameters as those used for Malvaceae. The *Theobroma* crown node calibration points were adjusted by limiting them to 10.11 Ma with a normal prior and stdev =  4. Six BEAST runs were conducted per 200,000,000 generations each, while parameters were sampled every 1,000 generations.

## Results

### Plastomic features of 19 *T. cacao* sequences

**Fig 1:**
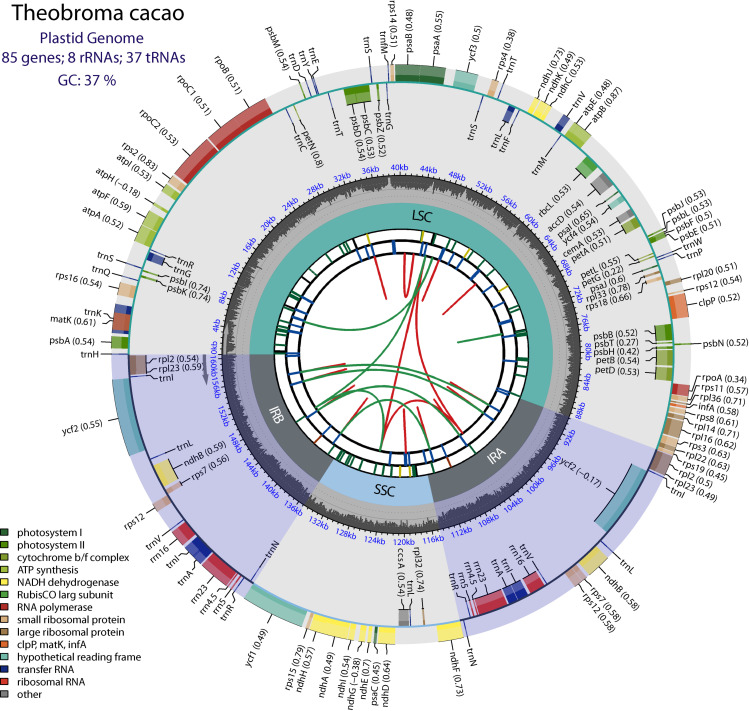
Circular genetic map of the general characteristics of the chloroplast genome of *T. cacao.* The map contains six default tracks. From the center outward, the first track shows the scattered repeats connected with arcs. The second track shows the long tandem repeats as short bars. The third track shows short tandem repeats or microsatellite sequences as short bars. The small single-copy (SSC), inverted repeat (IRa and IRb) and large single-copy (LSC) regions are shown in the fourth track. The GC content in the genome is represented in the fifth track. The genes are shown in the sixth track. The optional codon usage bias is shown in parentheses after the gene name. Genes are coded according to their functional classification. The transcription directions of the inner and outer genes are clockwise and counterclockwise, respectively. The functional classification of the genes is shown in the lower left corner.

In this study, the chloroplast genomes of seven *T. cacao* specimens were sequenced. Illumina single-end sequencing revealed a total of 2.23 ×  10^6^, 2.14 ×  10^6^, 2.98 ×  10^6^, 2.26 ×  10^6^, 2.21 ×  10^6^, 2.42 ×  10^6^ and 2.22 ×  10^6^ 150 bp reads for each sample, including INDES06, INDES14, INDES34, INDES50, INDES63, INDES67, and INDES71 with an average sequencing depth of 1,570.7; 973.7; 488.34; 1,161.2; 990.4; 203.26 and 990.1 respectively (S2 Fig). On average, 160 Mb of high–quality sequence was obtained from each specimen. Illumina sequencing of plastid DNA produced between 2,142,060 to 2,998,427 clean reads (150 bp) for the seven *T. cacao* samples analyzed. Seven full plastid genomes were obtained through assembly and annotation. The genomes of these angiosperms exhibit a typical genomic structure, as shown in Fig 1. The genes ranged from 160,589 to 160,727 bp in length and had a GC percentage of 36.9 (Table 3). The gene content comprises 130 genes, including 37 tRNAs, 8 rRNAs, and 85 protein-coding genes (Table 3). The inverted repetitive region (IR) contained 17 duplicated genes, six of which were protein–coding (four rRNA and seven tRNA). Furthermore, Table 4 outlines 22 genes associated with photosynthesis, eight genes associated with proton exchange, and 18 genes linked to electron exchange.

**Table 3:** Characteristics of complete chloroplast genomes of *T. cacao.*

Specie name	GenBank	Size (base pair; bp)				Number of genes					G ^+ ^ C (%)			Protein coding part (CDS) (%bp)
		Genome	LSC	SSC	IR	Total genes	Duplicate genes	CDS	tRNA	rRNA	Genome	CDS	All gene	
***T. cacao* (INDES06)**	**OP354232**	160,679	89,395	20,186	25,556	130	17	85	37	8	36.9	37.9	39.5	49.08
***T. cacao* (INDES14)**	**OP354233**	160,613	89,429	20,220	25,515	130	17	85	37	8	36.9	37.9	39.5	49.08
***T. cacao* (INDES34)**	**MZ725364**	160,620	89,407	20,183	25,515	130	17	85	37	8	36.9	37.9	39.5	49.08
***T. cacao* (INDES63)**	**OP354235**	160,613	89,293	20,188	25,516	130	17	85	37	8	36.9	37.9	39.5	49.08
***T. cacao* (INDES50)**	**OP354234**	160,727	89,333	20,187	25,511	130	17	85	37	8	36.9	37.9	39.5	49.08
***T. cacao* (INDES67)**	**MZ725365**	160,617	89,410	20,157	25,525	130	17	85	37	8	36.9	37.9	39.5	49.08
***T. cacao* (INDES71)**	**OP354236**	160,647	89,393	20,194	25,546	130	17	85	37	8	36.9	37.9	39.5	49.08
*T. cacao*	JQ228389	160,619	89,393	20,194	25,546	125*,1	17	82*,1	36*,1	8	36.9	37.9	39.5	49.08
*T. cacao*	JQ228380	160,619	89,333	20,194	25,546	125*,1	17	82*,1	36*,1	8	36.9	37.9	39.5	49.08
*T. cacao*	JQ228381	160,619	89,333	20,194	25,546	125*,1	17	82*,1	36*,1	8	36.9	37.9	39.5	49.08
*T. cacao*	HQ244500	160,619	89,393	20,194	25,546	125*,1	17	82*,1	36*,1	8	36.9	37.9	39.5	49.08
*T. cacao*	KY085907	160,619	89,393	20,194	25,546	125*,1	17	82*,1	36*,1	8	36.9	37.9	39.5	49.08
*T. cacao*	JQ228382	160,619	89,393	20,194	25,546	125*,1	17	82*,1	36*,1	8	36.9	37.9	39.5	49.08
*T. cacao*	JQ228383	160,619	89,410	20,194	25,583	125*,1	17	82*,1	36*,1	8	36.9	37.9	39.5	49.08
*T. cacao*	JQ228387	160,619	89,393	20,194	25,546	125*,1	17	82*,1	36*,1	8	36.9	37.9	39.5	49.08
*T. cacao*	JQ228386	160,619	89,393	20,194	25,546	125*,1	17	82*,1	36*,1	8	36.9	37.9	39.5	49.08
*T. cacao*	JQ228385	160,619	89,393	20,194	25,546	125*,1	17	82*,1	36*,1	8	36.9	37.9	39.5	49.08
*T. cacao*	JQ228379	160,619	89,393	20,194	25,546	125*,1	17	82*,1	36*,1	8	36.9	37.9	39.5	49.08
*T. cacao*	HQ336404	160,604	89,293	20,188	25,546	130	17	85	37	8	36.9	37.9	39.5	49.08
*T. grandiflorum*	JQ228388	160619	89,393	20,194	25,546	130	17	85	37	8	36.8	37.9	39.5	48.96

*Unannotated genes: *trnG, psbZ, rpl22, rpl2*(copy).

1Absence of the gene *infA*

**Table 4:** Genes encoded in the chloroplast genomes of *T. cacao.*

Category	Gene groups	Gene name
Photosynthesis	*Subunits of atp synthase*	*atpA, atpB, atpE, atpF, atpH, atpI*
	Subunits of NADH-dehydrogenase	*ndhA, ndhB* (x2)*, ndhC, ndhD, ndhE, ndhF, ndhG, ndhH, ndhI, ndhJ, ndhK*
	Subunits of cytochrome b/f complex	*petA, petB, petD, petG, petL, petN*
	Subunits of photosystem I	*psaA, psaB, psaC, psaI, psaJ*
	Subunits of photosystem II	*psbA, psbB, psbC, psbD, psbE, psbF, psbH, psbI, psbJ, psbK, psbL, psbM, psbN, psbT, psbZ, ycf3*
	Subunit of Rubisco	*rbcL*
Self-replication	Large subunit of ribosome	*rpl14, rpl16, rpl20, rpl22, rpl23* (x2)*, rpl2* (x2)*, rpl32, rpl33, rpl36*
	Small subunit of ribosome	*rps11, rps12* (x2)*, rps14, rps15, rps16, rps18, rps19, rps2, rps3, rps4, rps7* (x2)*, rps8*
	DNA dependent RNA polymerase	*rpoA, rpoB, rpoC1, rpoC2*
	Ribosomal RNAs	*rrn16* (x2)*, rrn23* (x2)*, rrn4.5* (x2)*, rrn5* (x2)
	Transfer RNAs	*trnH-GUG, trnK-UUU, trnQ-UUG, trnS-GCU, trnG-GCC, trnR-UCU, trnC-GCA, trnD-GUC, trnY-GUA, trnE-UUC, trnT-GGU, trnS-UGA, trnG-GCC* (x2)*, trnfM-CAU, trnS-GGA, trnT-UGU, trnL-UAA, trnF-GAA, trnV-UAC, trnM-CAU, trnW-CCA, trnP-UGG, trnI-CAU, trnL-CAA, trnV-GAC, trnI-GAU, trnA-UGC, trnR-ACG, trnN-GUU, trnL-UAG, trnN-GUU* (x2)*, trnR-ACG* (x2)*, trnA-UGC* (x2)*, trnI-GAU* (x2)*, trnV-GAC* (x2)*, trnL-CAA* (x2)*, trnI-CAU* (x2)
Other genes	C-type cytochrom synthesis gene	*ccsA*
	Envelop membrane protein	*cemA*
	Maturase	*matK*
	Protease	*clpP*
	Subunit of Acetyl-CoA-carboxylase	*accD*
	Translational initiation factor	*infA*
Genes of unknown	Conserved open reading frames	*ycf1, ycf2* (X2)*, ycf4*
Possible missing protein coding genes	Conserved open reading frames	*ycf15*
Genes with nonstandard start codon	GAT	*psbI*
	ATT	*infA*
	ACG	*ndhD*

### Simple sequence repeats and dispersed repeats

The number of simple sequence repeats (SSRs) found in the 19 chloroplast genomes of *T. cacao* ranged between 73 and 80. A and T were determined to be the most common SSRs, with no G-type mononucleotides present in any of the *T. cacao* samples (S3A Fig). The most prevalent SSR in terms of the frequency of classified repeat types (in relation to the complementary sequence) was A/T-type mononucleotide (S3B Fig). The chloroplast genomes exhibited diverse types of SSRs, the most prevalent of which were single nucleotide repeats, which occurred 64–67 times. In contrast, dinucleotide repeats appeared only six times, followed by trinucleotide and pentanucleotide repeats once and one to two times, respectively. Notably, no tetranucleotide repeats were detected (S3C Fig). In the specified length intervals (30–39, 40–49, 50–59, 60–69 and ≥  70), the most abundant SSRs were between 30 and 39 nucleotides in length, followed by those 50–59 in length. The ranges of 40–49, 60–69 and ≥  70 exhibited the fewest SSRs, as illustrated in S3D Fig. The most prevalent types across all samples were repeats and palindromic repeats, whereas complementary repeats were the least frequent.

### Inverted-repeat contraction, expansion, and interspecific comparison

**Fig 2:**
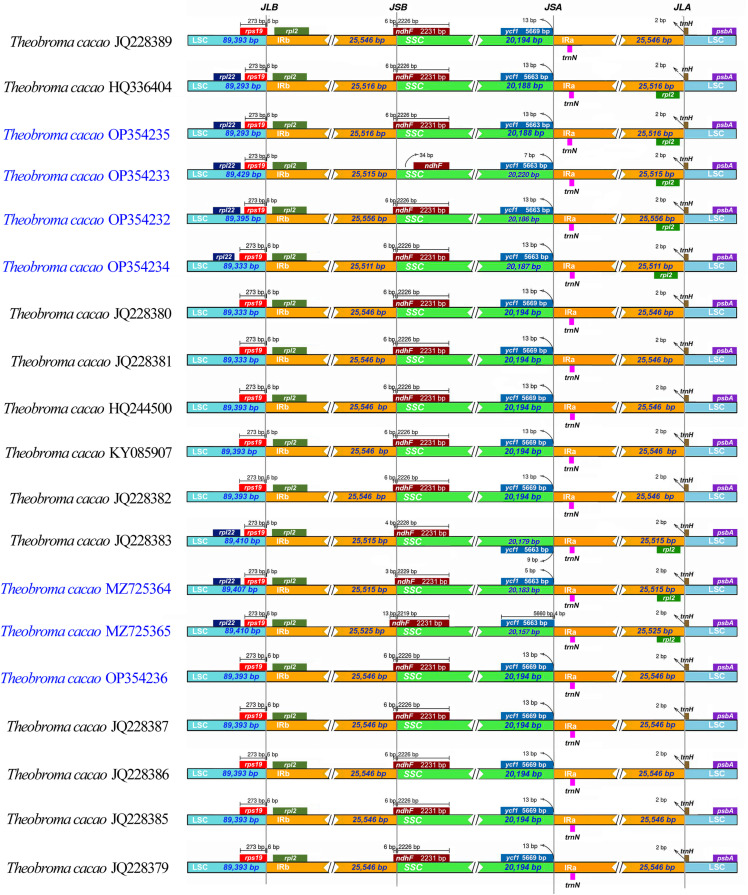
A comparison of large single-copy (LSC), inverted repeat (IR), and small single-copy (SSC) junction positions was conducted across 19 *T. cacao* plastomes. The distance to the boundary or the length of genes in single-copy regions and IR regions is indicated next to each gene.

We analyzed the junctions of the inverted repeat (IR) region and the two single–copy regions in the 19 *Theobroma* genomes, which included the 7 genomes examined in this study, as well as the adjacent gene locations (Fig 2). The long single-copy (LSC), IR, and short single-copy (SSC) regions had comparable lengths. The genes located at the junction sites consisted of *rpl22, rps19, rpl2, ndhF*, *ycf1*, *trnN, trnH*, and *psbA*. Although the *rpl22* gene was present in the LSC region, it was detected in only 8 of the genomes. The *rps19* gene was identified at the junction of the LSC and IRb sections, while the *rpl2* gene was located solely within the IRa and IRb regions and was detected in just 8 genomes. The *ndhF* gene was detected within the SSC and IRb regions at 3 to 6 bp intervals, except for the INDES14 (KUELAP-219) genome, where it was exclusively located in the IRb region. Similarly, the *ycf*1 gene is typically found within the SSC region, but in the case of the INDES67 (KUELAP-659) genome, it was identified in both the SSC and IRa regions, with only a 4 bp gap between them (Fig 2). The *trnN* gene was fully located within the IRa region in all 19 genomes. The *trnH* gene was located in the LSC region and crosses the boundary of 2 bp in the IRa region. Moreover, the *psbA* gene was detected in the LSC region in all 19 genomes (Fig 2). Additionally, 12 cis-splicing and one trans-splicing genes were identified in all genomes.

### Polymorphism analysis of *T. cacao* chloroplast genomes

**Fig 3:**
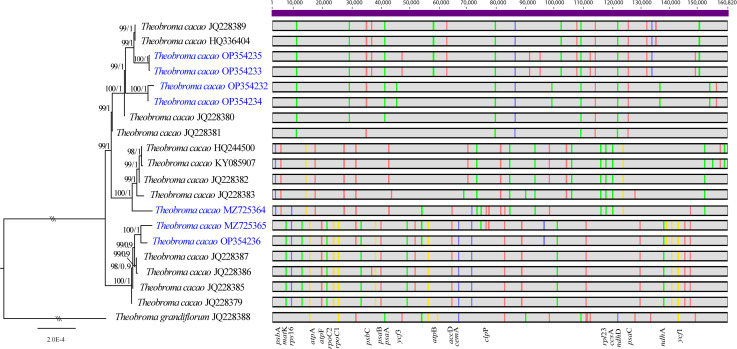
Phylogenomic tree of *T. cacao* generated by maximum likelihood inference. The nodes indicate bootstrap support (BS) and posterior probability (BPP) and are presented above the branches. The scale represents the number of nucleotide substitutions per site. The horizontal bars show whole genomes, and the colored vertical bars on each genome indicate SNPs.

A total of 80 polymorphic sites were identified in the chloroplast whole-genome sequences of 19 *T. cacao* genomes, among which were 56Indels and 58 singleton variants. The genes *matK, atpF, rpoC2*, *psbC, psaA, cemA, rpl32*, *ccsA, ndhD, psaC* and *ycf1* exhibited 14 variation sites in total (Fig 3). The genes *ycf1* (3 variants), *rpoC2* and *psbC* (each with 2 variants) had the highest number of variation sites. Moreover, these genes also had the greatest number of Indels (2 each). The intergenic regions that exhibited the most variation were found to be *rpl32-trnL*, *matK-rps16, nahF-rpl32*, *atpH-atpF*, and *rps15-yfc1* (Fig 3; S4 Fig). These variations, along with the use of *T. grandiflorum* as an outgroup, resulted in a tree with significantly high support values on each branch (Fig 3). The alignment of all the plastid genomes indicated a high degree of similarity (greater than 50% identity) in the total sequence, with intraspecific divergence of 0.0006 and 0.04%, respectively. The interspecific divergence between *T. grandiflorum* and Fine Aroma Cocoa amounted to 0.28%. Furthermore, we identified three Fine Aroma Cocoa groups that exhibit comparable genomic characteristics. The first of these groups (BS/BPP =  99/1), contained samples INDES63 (OP354235), INDES14 (OP354233), INDES06 (OP354232), and INDES50 (OP354234). Collectively, these strains formed a sister clade to other *T. cacao* genomes (JQ228389, HQ336404, JQ228380, and JQ228381). A second clade, with a posterior probability/bootstrap value of 100/1, consisted of sample INDES34 (MZ725364). This sample was closely related to the JQ228382, HQ244500, JQ228383, and KY085907 genomes. The third clade, (with a posterior probability/bootstrap value of 100/1), included INDES67 (MZ725365), and INDES71 (OP354236). This clade was closely related to the cocoa genomes JQ228385, JQ228386, JQ228387, and JQ228379 (Fig 3).

### Phylogenetic analysis

**Fig 4:**
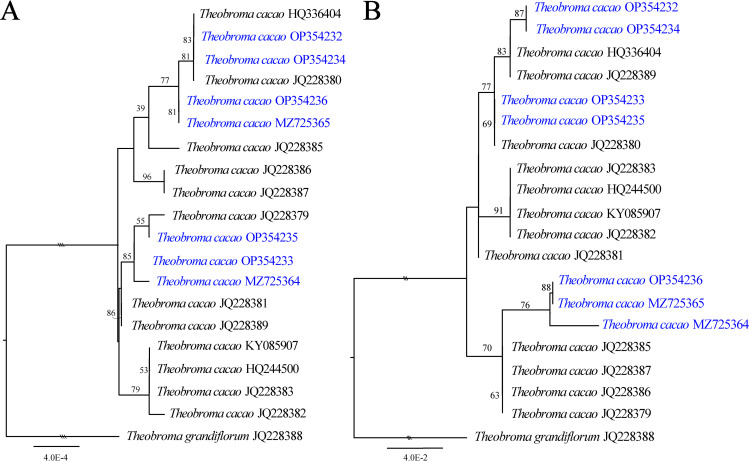
Maximum-likelihood phylogenetic inference of 19 *T. cacao* species based on the *matK* + *infA* + *ycf1* genes (A) and the *rpl32*–*trnL* + *matK–rps16* + *nahF–rpl32* spacers (B). The numbers associated with the nodes are bootstrap support (BS) values. The scale indicates the number of nucleotide substitutions per site.

The tree topology based on the whole *T. cacao* genome was shown to be identical to that of three genes (*matK*, *infA*, and *ycf1*) as well as three intergenic regions (*rpl32*-*trnL*, *matK*-*rps16*, and *nahF*-*rpl32*) (Fig 4A and B). The *rpl32-trnL* spacer (S4A Fig) and the *ycf1* gene located in the SSC region (S4D Fig) were found to be the most appropriate regions for uniform topology on an independent basis. This ability is determined by analyzing the complete sequence of the plastid genomes and considering the exons and introns. On the other hand, the *matK*-*rps16* (S4B Fig) and *nahF*-*rpl32* (S4C Fig) spacers, as well as the *matK* and *infA* genes, exhibited low topology similarity (S4E and F Fig). The intraspecific differentiation rate of the *matK* +  *infA* +  *ycf1* combination was 1%, which included coding regions of 9,438 bp and noncoding regions of 4,805 bp, demonstrating noteworthy similarity, with an identity greater than 50%. The *ycf1* and *matK* genes exhibited divergence rates of 0.01–0.9% and 0.03–0.1%, respectively. Nevertheless, the *infA* coding region presented a high degree of intraspecific divergence (38%) due to a deletion of 80 bp in some genomes, leading to nonrecognition of this *infA* gene (S5 Fig). The combination of noncoding regions showed intraspecific divergence ranging from 0.04–0.2%. Additionally, the spacer sequences of *rpl32-trnL, matK-rps16,* and *nahF-rpl32* exhibited divergence rates of 0.08–0.2%, 0.07–0.1%, and 0.09–0.5%, respectively.

### Estimation of *Theobroma* divergence time

**Fig 5:**
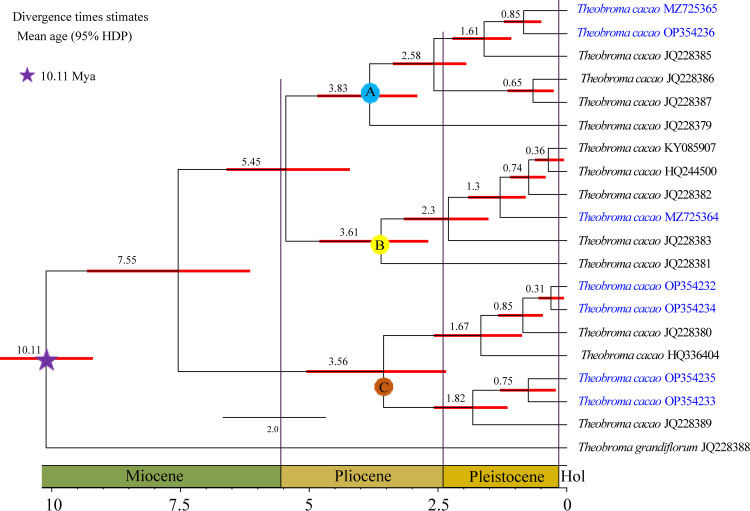
*Theobroma* chronogram based on protein coding sequences estimated from BEAST. The values at the nodes indicate divergence dates in millions of years.

The age of the Malvaceae crown node was estimated to be 70.7 Ma, while that of the *Theobroma* stem was 52.4 million years (S6 Fig). The node age of *T. cacao* and *T. grandiflorum* was estimated to be 10.11 million years ago (Fig 5). These estimations suggest that the 19 species of *T. cacao* had a common ancestor approximately 7.55 million years ago (95% HPD), diverging into three clades approximately 3.83 million years ago (95% HPD) (A), 3.61 million years ago (95% HPD) (B), and 3.56 million years ago (95% HPD) (C). From this time onwards, many species began to undergo independent evolution during the Pleistocene epoch, which lasted from approximately 0.31 to 1.82 million years (Fig 5). Samples INDES67 (MZ725365) and INDES71(OP354236) shared a common ancestor estimated to have lived approximately 850,000 years ago. Samples INDES06 (OP354232) and INDES50 (OP354234) similarly shared a common ancestor approximately 310,000 years ago. In addition, it is estimated that samples INDES63 (OP354235) and INDES14 (OP354233) shared a common ancestor approximately 750,000 years ago, while INDES34 (MZ725364) appeared approximately 1.3 million years ago (Fig 5).

## Discussion

### Chloroplast genome structure

In this study, the plastidial genomes of Fine Aroma Cocoa were decoded. This is the first study in the Amazonas region in which massive sequencing technologies have been used to locate and assign functions to plastid genes in this important crop. The structure, content, organization, and characteristics of the plastid genomes of seven Fine Aroma Cocoa samples (INDES06, INDES14, INDES34, INDES50, INDES63, INDES67, and INDES71) demonstrated significant similarity to other *Theobroma* plastid genomes, including those of *T. grandiflorum* [20], as well as to plastid genomes of *Gossypium* [59], *Tilia* [60,61], and *Hibiscus* [62]. However, there were apparent variations in the sizes of the LSC, SSC, and IR regions (Fig 2, Table 3). These differences indicate that the IR regions are more stable within *T. cacao*, a phenomenon that prevails throughout other Malvales species [56]. Although there were considerable increases in the RI and LSC boundaries within *Gossypium* [59], *Tilia* [59,61], and *Hibiscus* [62], these expansions were modest. These modifications to the IR regions could be linked to the formation of pseudogenes, similar to what occurs in Malpighiales [63].

### Simple sequence repeats and dispersed repeat contents

Our findings showed that *Theobroma* sequences exhibit comparable GC contents [13,22]. All the genomes shared similar properties, including a total number of genes (130), duplicated genes (17), and protein-coding genes (85), with the exception of the *infA* gene, which functions in *T. cacao* and other genera of Malvaceae, such as *Tilia* [60,61]. However, in *T. grandiflorum*, its function has yet to be determined [22], and in other nearby genera, such as *Gossypium* [59], *Hibiscus* [22,62], and *Sida* [64], the *infA* gene functions as a pseudogene. The *infA* gene was identified in seven examined genomes in this study (S5 Fig). The *infA* gene functions to regulate the selection of mRNA, creating the preinitiation complex. Nevertheless, the gene is absent in other published cocoa genomes because of an 80-base pair loss, which renders it unrecognizable (S5 Fig). This finding suggested that the gene may have undergone an evolutionary event, been transferred, or been functionally replaced by another gene in the nucleus. This hypothesis is supported by nuclear transcriptome analysis, which revealed genetic transfers, such as *infA* and *rpl32*, from the chloroplast to the nucleus in *Hypericum ascyron* [65]. Moreover, the research conducted by Park et al. [66] and Millen et al. [67] on *Thalictrum coreanum* and other angiosperms, respectively, demonstrated evolutionary variations that suggest such gene transfers are possible. A majority of genes contain an AUG initiation codon, except for GTG [68]. However, according to the results of the present study, the *infA* gene harbors a UUG initiation codon, which is effective at precisely initiating the translation of *infA* mRNA [65,67], despite its inefficiency as an initiation codon [68]. However, further studies on the nuclear transcriptome of *T. cacao* species are necessary to elucidate the evolutionary changes in the *infA* gene. It is also crucial to investigate other unknown functions within the plastid genome [22]. Additionally, it is essential to locate other genes that are typically present more frequently in subtelomeric regions in cacao [13,14]. It has been determined that codons with the same terminus (A/T) are responsible for encoding most amino acids. This trend is also observed in genomes with high AT percentages, which are typical of Malvaceae species [13,22,56]. One potential reason for the increased frequency of A/T repeats is polyadenylation at the mRNA end in the cp genes of various species [61]. In addition, during plastome replication, strand separation is easier for A/T pairs than for G/C pairs [69]. Therefore, the simple sequence repeats (SSRs) identified in this study will be beneficial for future population genetics and evolutionary investigations of Fine Aroma Cocoa. Furthermore, these SSRs are important sources of molecular markers for biogeographic research [70,71].

### Polymorphism analysis of *T. cacao* chloroplast genomes

The variation in size observed in each plastid genome of Fine Aroma Cocoa is attributed to the accumulation of indels, which results in genetic variation [22,72]. Additionally, this genetic variation could have arisen from virus–derived eukaryotic genes, prompting genetic material exchange and early diversification in plastidial and mitochondrial genomes through glycosyltransferases [73], viruses use genes from their hosts to replicate and spread throughout plants [74,75], thereby overcoming the host immune system and becoming crucial assets for adaptation [76,77], and evolution [78,79]. While some of these genes are conserved, others serve novel functions in plants [80,81]. However, this hypothesis has not yet been tested in cacao plantations in Amazonas, as Bustamante et al. [31] reported that the majority of cacao samples from the Amazonas region analyzed by SNPs were heterozygous, meaning that the coexistence of multiple alleles within plant cells leads to high levels of heterogeneity in the products of plastid genome copies, resulting in the occurrence of transposable elements (TEs) [82–85], as occurs in species of *Gossypium* [59], *Tilia* [60,61] and *Hibiscus* [62]. This suggests that the Fine Aroma Cocoa plantations exhibit genetic variability due to successive backcrossing of trees, along with various insertions or deletions within the plastidial genomes caused by environmental factors, resulting in polymorphisms that can persist across generations of populations. The genetic material of plantations with low homozygosity is relatively conserved [31]. Sequence differences were detected in the exon and intronic regions of each genome, suggesting the potential use of intraspecific SNPs in identifying differential allele expression, including interspecific hybrid expression studies [86]. Given that every haplotype has a unique cpSNP profile, we can distinguish genetic clusters within the cacao population [19]. For instance, *trnH-psbA* chloroplast region SNPs are utilized as markers to identify cacao haplotypes [87]. However, our study revealed that the *ycf1* gene has a greater number of SNPs than other genes and could be a more efficient method for assessing intraspecific genetic variability in Fine Aroma Cocoa. This is due to the *ycf1* gene having both SNPs and Indels in coding sequences. Additionally, other genes, such as *infA* and *rpoC2,* were described in the present study; however, these genes were insufficient for distinguishing genetic groups within Fine Aroma Cocoa. Alternatively, paternal transmission (paternal leakage) of chloroplasts through pollen [83] may also contribute to the variation of repetitive simple sequences or the presence of indels in some varieties of Fine Aroma Cocoa. Nevertheless, the hypothesis has yet to be subjected to rigorous examination in the context of cocoa plants, underscoring the necessity for further research to elucidate the phenomenon of autocopatibility. Future studies will concentrate on identifying viral sequences integrated into the cacao genome to determine possible pathways associated with probable the horizontal gene transfer (HGT) and to investigate the existence of heteroplasmy and/or polyplasmy. since, genetic heterogeneity of a few homozygous trees prevalent in Fine Aroma Cocoa on various plantations in Amazonas region [31].

### Phylogenomic analysis and search for specific genes

Phylogenomic analysis has shown that the plastid genomes INDES71 (OP354236), INDES67 (MZ725365) (Fig 5; Clade A) are closely related to the genetic group previously identified as Trinitarios and cacao criollo pure [45], whose samples were collected in Suriname, Trinidad and Tobago and the United States [11,88]. However, the study carried out by Bustamante et al. [31] produced different results, revealing that Amazonas cocoa shares genetic material with almost all the varieties previously described by Motamayor et al. [11], where one variety usually predominates over the others. For example, of the two samples INDES71 (OP354236) and INDES67 (MZ725365), the INDES67 sample exhibited the highest genetic loads for the National (33) and Contamana (26.21%) varieties [31]; the INDES71 sample was generated by combining 46.05% of the Iquitos variety and 39.25% of the National variety, as reported by Bustamante et al. [31]. On the other hand, the INDES34 sample was grouped in clade B with varieties of Trinitarios and Forasteros from the Lower Amazonas. Finally, four of the seven plastid genomes examined within clade C in this study display resemblances to outsider samples from the Lower Amazonas. Although the primary genetic variations present in INDES06 (OP354232) were National (68%) and Iquitos (12.46%), in INDES14 (OP354233) and INDES63 (OP354235), National (81.68% and 75.69%) and Curaray (17.49% and 19.65%) were more prevalent. However, the clarity of the data is limited. In the INDES50 (OP354234) dataset, the National (36.02%) and Amelonado (13.8%) genetic varieties are prevalent [31]. To determine the predominant genetic group in these samples, the original material must be evaluated. The distinction between genetic groups indicates significant interbreeding and gene flow in cocoa [45]. Hence, it is crucial to conduct research at the genomic level to comprehend the diversity of Fine Aroma Cocoa. Even if two varieties share a recent ancestor in common, distinct differences can still be identified and demonstrated [45].

Furthermore, to clarify the complex connection between *T. cacao* species, inter- or intraspecific analysis requires specific or universal markers. Therefore, the chosen barcode should be both variable and conserved to facilitate successful design, PCR amplification, and sequencing [89]. The initial plant barcodes selected were *rbcL* and *matK* [90], with *rbcL* being deemed optimal for lower plants [89] and *matK* being deemed optimal for angiosperms [91]. Other commonly utilized regions of the plant molecular systematics plastid genome include *atpF-H, psbK-I, ropC1, rpoB, trnH-psbA*, and *trnL*-*F* [92–95]. However, this study revealed these regions to be ineffective at distinguishing genetic groups within *T. cacao*. These methods may be more advantageous for genus-level classifications within the Malvaceae. For instance, *trnH-psbA* exhibited poor universal marker efficiency due to its variability across all plastid genomes [96]. Its variability exceeds that of *matK* and *rbcL* [97]. However, its use as a universal barcode is limited by inversion and insertion [89]. This phenomenon was also observed in the other regions examined in this study, namely, *rpl32-trnL, matK-rps16, nahF-rpl3, trnS-G, accD-psaI, atpF-H, psbK-I, ropC1, rpoB,* and *trnL-F*. In addition, these regions were not useful for distinguishing between organisms of the same *T. cacao* species, whereas combinations of several intergenic regions, such as *rpl32-trnL, matK-rps16,* and *nahF-rpl3*, were among the other combinations and proved to be more effective in differentiating organisms within *T. cacao* (Fig 4). Thus, the potential of using these markers in combination to distinguish among groups of *T. cacao* cannot be ignored, as the amalgamation of these regions proves to be more practical for distinguishing individuals of separate species [92–95].

In contrast, previous studies have suggested that *ycf1* and *ndhF* provide valuable data for DNA barcoding owing to high levels of variation in flowering plants [90,98]. This study established that the entire *ycf1* gene sequence enables the differentiation of individuals within the same species of *T. cacao* more effectively than combinations of the *matK* +  *infA* +  *ycf1* genes (Fig 4A) and the *rpl32-trnL* +  *matK-rps16* +  *nahF-rpl32* spacers (Fig 4B). These findings suggest that the *ycf1* gene can function as a universal marker for demarcating species within Malvaceae and other plant groups, similar to its use in several phylogenetic applications for Pinaceae [99], Orchidaceae [100], Lamiaceae [101], and *Prunus* [98]. It has also been successful in studies of several angiosperms, gymnosperms, monilophytes and bryophytes [102]. The *ycf1* gene is functional and essential for plant viability because it acts as a protein precursor and is not usually lost [103]. However, its application might not be useful in all taxa [104] due to the absence of the *ycf1* gene in Poaceae species [89].

### Estimation of *T. cacao* divergence time

Recent research has indicated that Fine Aroma Cocoa plants are genetically descended from the National variety, with some genetic contributions from Criollo and other varieties [31]. However, the genetic differentiation of these cacao plants has not been determined. Using seventy-eight coding sequences of complete plastid genomes, this study estimated the divergence time of Malvaceae to be approximately 70.7 Ma, which is in agreement with the results of Wang et al. [56]. Although there are certain limitations in the methods of analyzing and sampling taxa, as noted by Wang et al. [56], the results of this study are consistent with our ability to calculate the age of diversification of *T. cacao* (7.55 Ma), suggesting that this economically important species has had ample time to generate significant within species genetic diversity [105]. It can be deduced that between 3.5 and 3.9 Ma, three ancient and distinct lineages emerged and dispersed into cacao populations (clades A, B and C; Fig 5). These populations resulted in the majority of the samples analyzed in this study, and they originated in a recent Pleistocene era (0.31–2.3 Ma), with the exception of samples JQ228379 and JQ228381, which date back to the Pliocene era (approximately 3.61–3.83 Ma); it is likely that cocoa populations diverged during the Pliocene or Miocene epochs. Other contemporary populations may have adapted during the Holocene era, which was the most recent epoch of the Quaternary period. The separation of the three now extinct lineages (clades A, B, and C, as depicted in Fig 5) does not necessarily imply that the current individuals are pure. This study confirmed that the samples analyzed exhibited a certain degree of genetic material from the various genetic groups previously described by Motamayor et al. [11]. This finding supports the hypothesis proposed by Bustamante et al. [31] that a significant portion of Fine Aroma Cocoa plants are heterozygous, with a relatively small number of homozygous individuals. Evidence of this evolutionary process includes the partial loss of the *infA* gene (S5 Fig) in published cocoa samples, while our analyzed samples contained the complete gene. In addition to the variability in insertions and deletions has also been detected within the plastid genome, as has been observed for the variability in evolutionary rate between genes and lineages in cotton chloroplasts [85]. Therefore, the possibility that these evolutionary phenomena occurred in Fine Aroma Cacao cannot be excluded, given that the genus *Theobroma* diversified at an accelerated rate within Malvaceae during the mid-Miocene Andean uplift [105].

## Conclusions

In this study, the plastid genomes of Fine Aroma Cocoa were decoded. This is an innovative application of large-scale sequencing technologies in Peru, which aids in the identification and analysis of plastid genes of this important crop in the Amazonas region. As a result, complete sequencing of the plastid genomes provides a more precise understanding of the intraspecific relationship of Fine Aroma Cocoa. These findings suggest that plastid genomes retain the common genomic structure of angiosperms, containing 130 genes, 37 tRNAs, 8 rRNAs, and 85 protein-coding genes, with a 36.9% GC content. The structure of the plastid genome also demonstrated notable evolutionary development, as the *infA* gene was present in all the samples analyzed, in contrast with published cacao plastid genomes. Furthermore, the complete sequence of the *ycf1* gene was found to hold more promise for studying intraspecific relationships in *T. cacao*. Finally, the estimated ages of the *T. cacao* and *T. grandiflorum* nodes date back to 10.11 million years ago (Ma). These approximations suggest that *T. cacao* diverged approximately 7.55 Ma, and it is highly probable that cacao populations diversified during the Pliocene or Miocene epochs. It is imperative to conduct mitochondrial and nuclear studies on a greater number of cocoa samples to determine the credibility of these evolutionary processes, including genetic estimates and divergence. This approach allows us to investigate the validity of the aforementioned processes.

## Supporting information

S1 Fig Map collections of the 7 trees of the *T. cacao* from the Region Amazonas, northern Peru. This map was created by the authors using open access resources. The national, provincial, and district boundaries were obtained from the Geoportal of the National Geographic Institute of Peru (IGN) in shapefile format with a DATUM WGS 1984, following link: https://www.idep.gob.pe/geovisor/VisorDeMapas-3D/, which is located within the spatial information MED: http://sigmed.minedu.gob.pe/descargas/ (accessed on 6 August 2023). The map is for illustrative purposes only.
(TIF)

Table S1 List of species used for divergence analysis of Malvaceae, including the new complete chloroplast genomes of *T. cacao.* (DOCX)

S2 Fig The sequencing depth map of the *T. cacao* chloroplast genome. a =  INDES06, b =  INDES14, c =  INDES34, d =  INDES50, e =  INDES63, f =  INDES67 and g =  INDES71. The depth of each base was calculated by samtools depth.
(TIF)

S3 Fig Distribution of SSRs and dispersed repeats in the chloroplast genomes of *T. cacao.* (A) Frequency of identified SSR motifs; (B) Frequency of classified repeat types (considering sequence complementary); (C) Numbers of different SSR types detected in the cp genomes; (D) Numbers of dispersed repeat types having a given length interval (30 to 39, 40 to 49, 50 to 59, 60 to 69 and ≥  70).
(TIF)

S4 Fig Maximum-likelihood phylogenetic inference of 21 *T. cacao* individuals based on the *rpl32_trnL* spacer (A), *matK*_*rps16* spacer (B), *ndhF*_*rpl*32 space (C), *ycf1* gene (D), *matK* gene (E) and *infA* gene (F). The numbers associated with the nodes are bootstrap support (BS) values. The scale indicates the number of nucleotide substitutions per site.
(TIF)

S5 Fig Comparison of the *inf*A gene in the different chloroplast genomes of *T. cacao*, including *Theobroma grandiflorum.* (TIF)

S6 Fig Chronogram of Malvales based on 78 CDSs sequences estimated from BEAST. The red and blue star represent two fossil constraints and the green star represents one secondary calibrations obtained from the literature.
(TIF)
