# Supplementary figures and images for: Comparative analyses of chloroplast genomes of Theobroma cacao from northern Peru
Source: PLoS One. 2025 Mar 5;20(3):e0316148. doi: 10.1371/journal.pone.0316148 (PMC11882073; doi:10.1371/journal.pone.0316148)

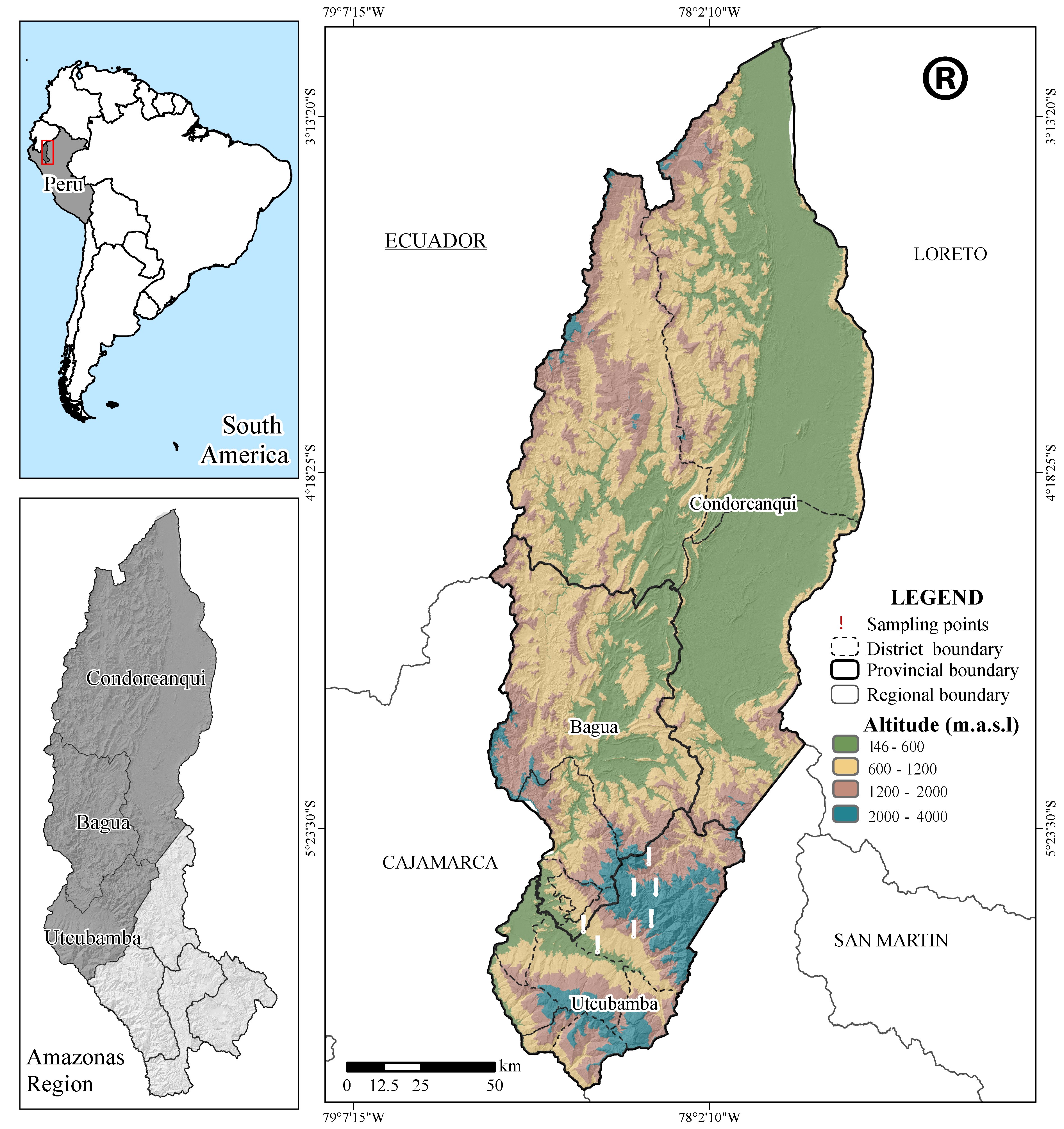

Supplement: S1 Fig — This map was created by the authors using open access resources. The national, provincial, and district boundaries were obtained from the Geoportal of the National Geographic Institute of Peru (IGN) in shapefile format with a DATUM WGS 1984, following link: https://www.idep.gob.pe/geovisor/VisorDeMapas-3D/, which is located within the spatial information MED: http://sigmed.minedu.gob.pe/descargas/ (accessed on 6 August 2023). The map is for illustrative purposes only. (TIF) [file pone.0316148.s001.tif]

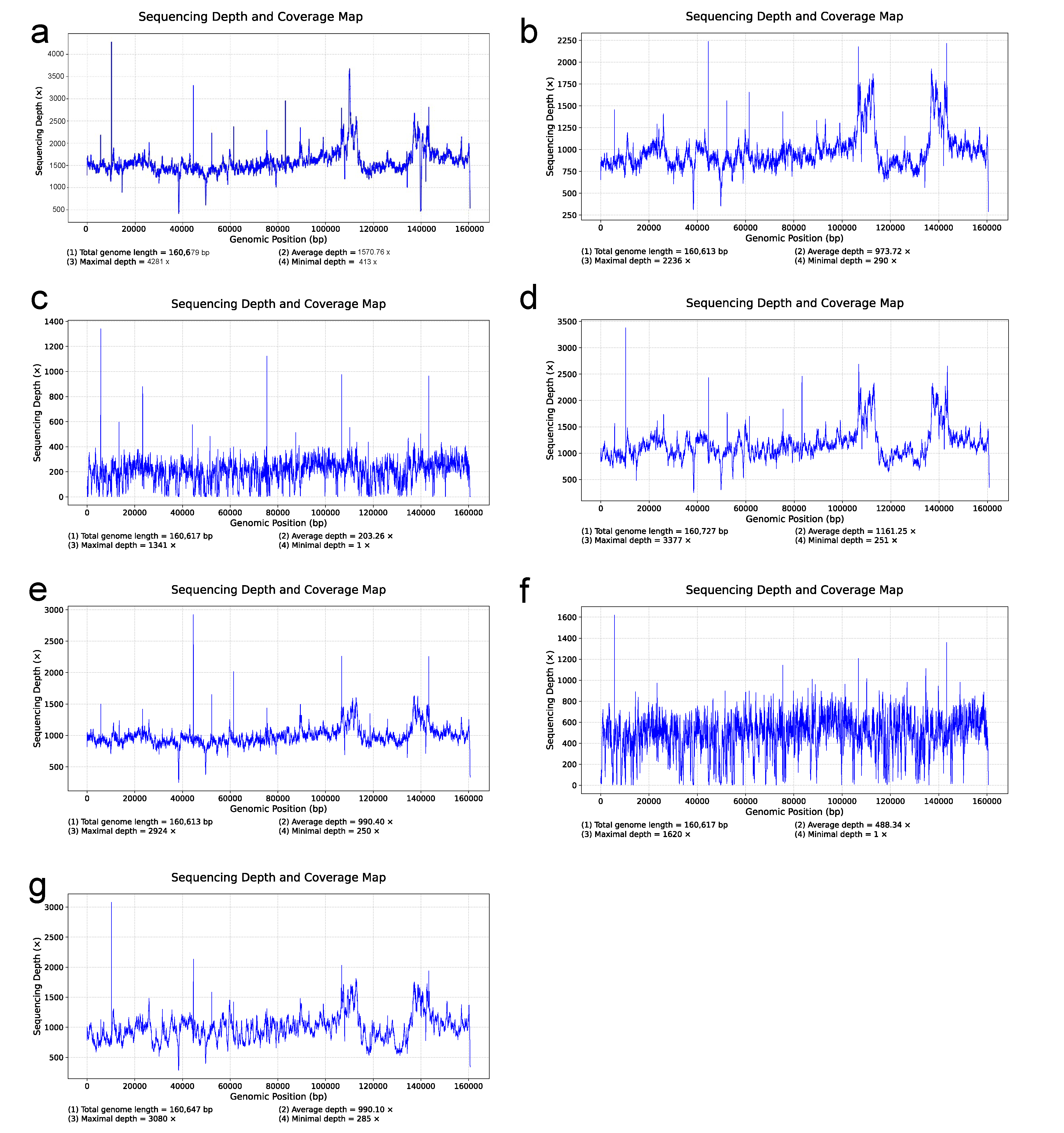

Supplement: S2 Fig — The depth of each base was calculated by samtools depth. (TIF) [file pone.0316148.s003.tif]

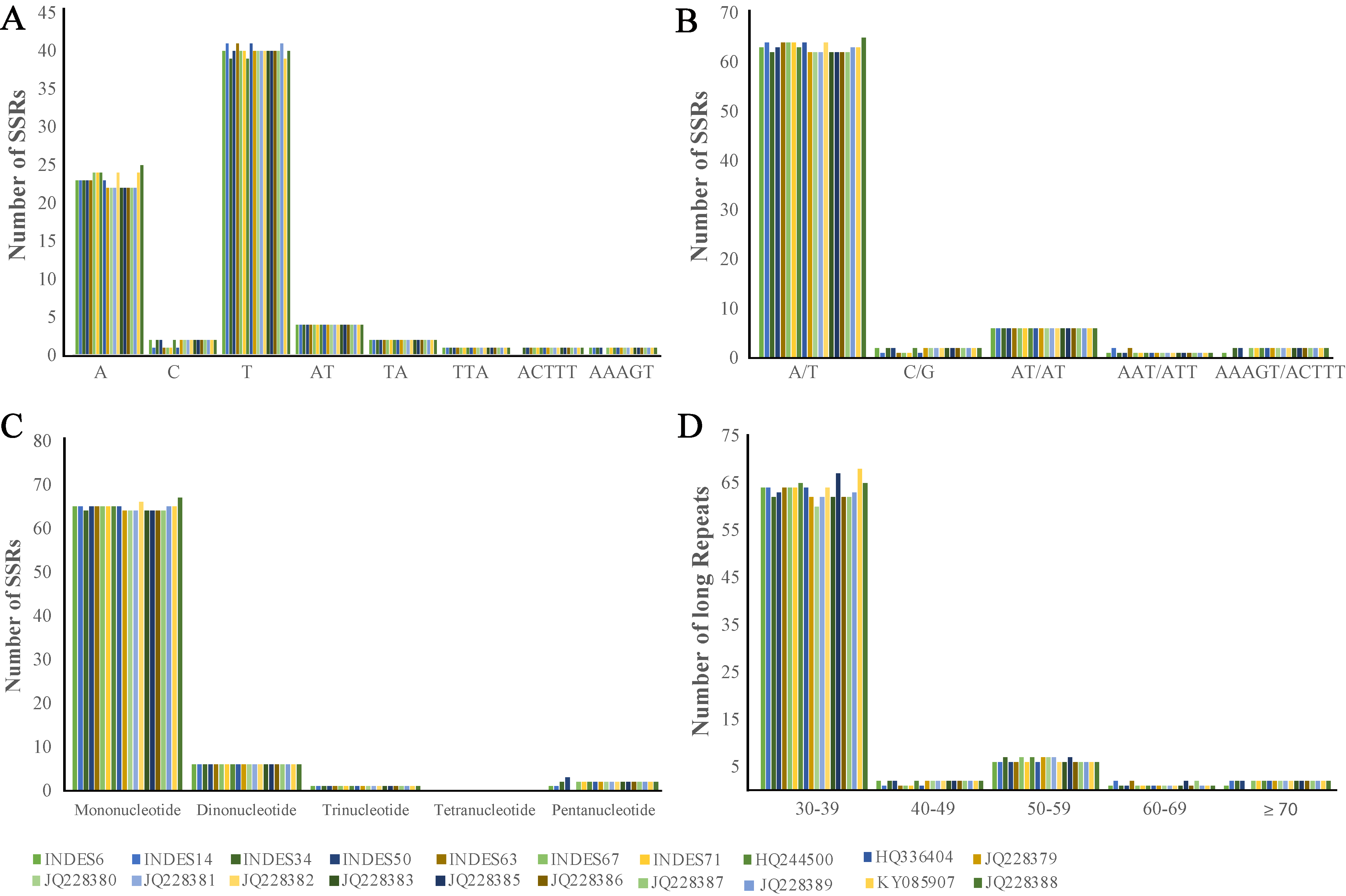

Supplement: S3 Fig — (A) Frequency of identified SSR motifs; (B) Frequency of classified repeat types (considering sequence complementary); (C) Numbers of different SSR types detected in the cp genomes; (D) Numbers of dispersed repeat types having a given length interval (30 to 39, 40 to 49, 50 to 59, 60 to 69 and ≥ 70). (TIF) [file pone.0316148.s004.tif]

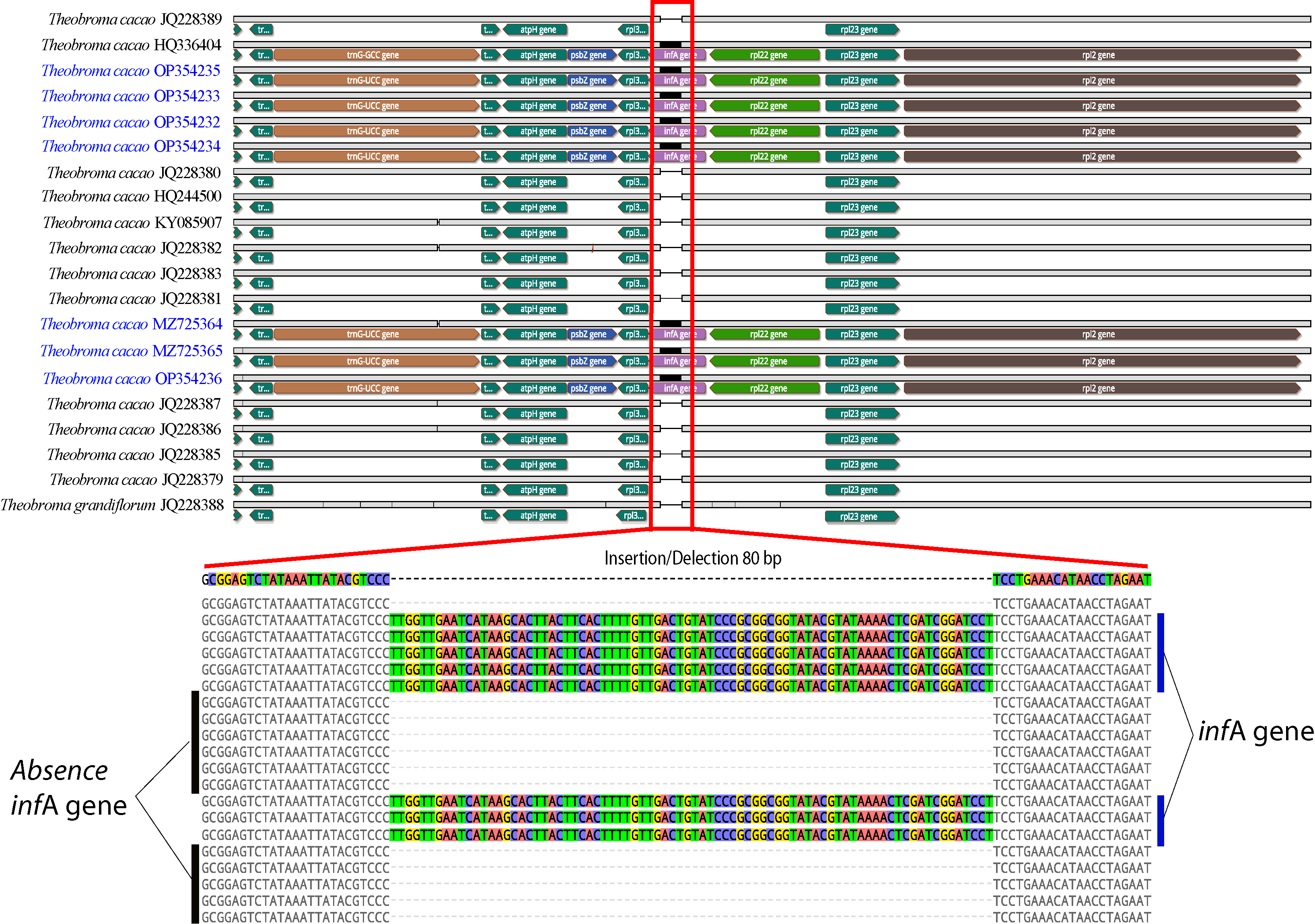

Supplement: S5 Fig — (TIF) [file pone.0316148.s006.tif]

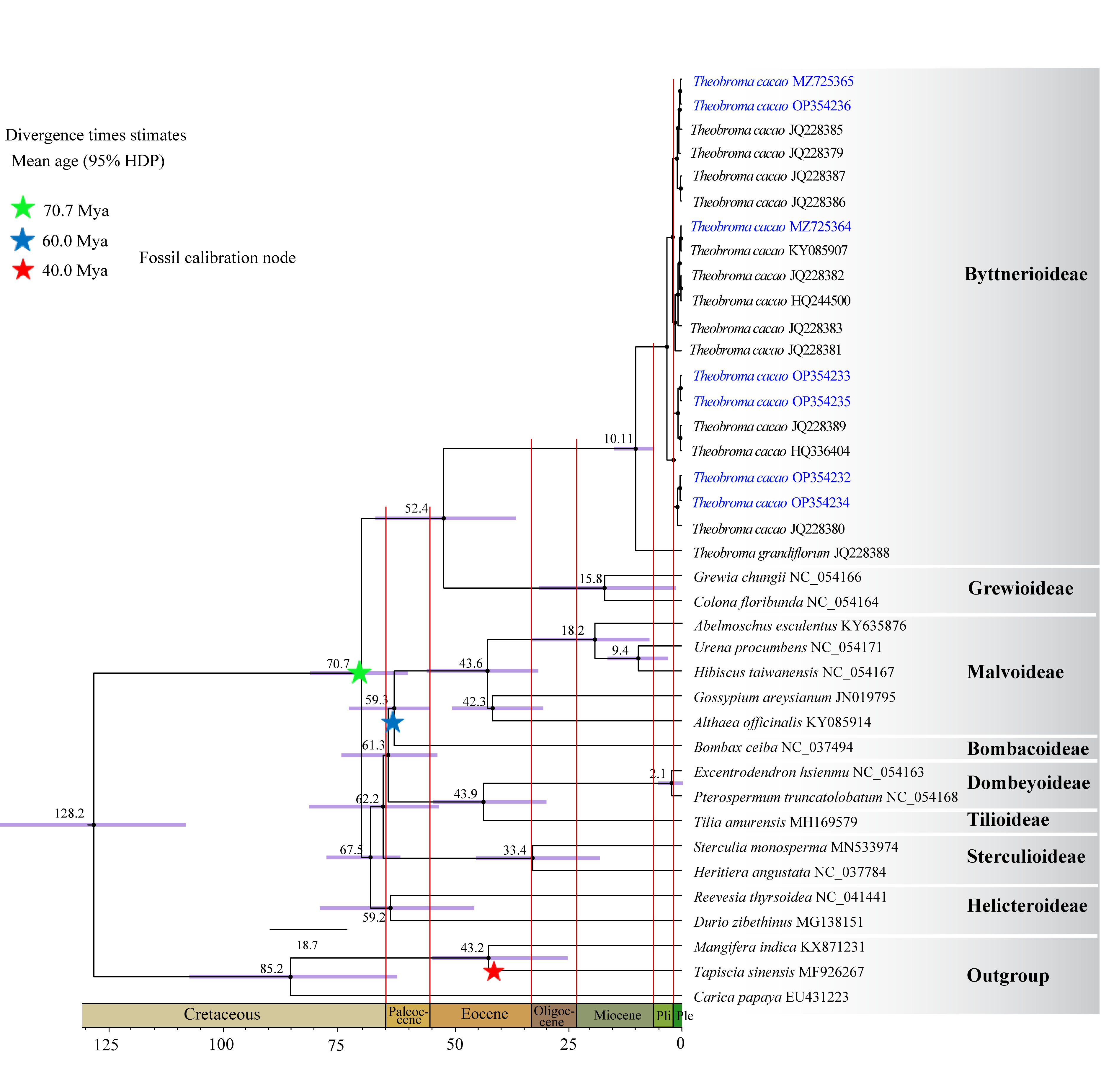

Supplement: S6 Fig — The red and blue star represent two fossil constraints and the green star represents one secondary calibrations obtained from the literature. (TIF) [file pone.0316148.s007.tif]
