# Supplementary material for: Comparative analyses of chloroplast genomes of Theobroma cacao from northern Peru
Source: PLoS One. 2025 Mar 5;20(3):e0316148. doi: 10.1371/journal.pone.0316148 (PMC11882073; doi:10.1371/journal.pone.0316148)
Supplement: Table S1 — (DOCX) [file pone.0316148.s002.docx]

**Table S1.** List of species used for divergence analysis of Malvaceae, including the new complete chloroplast genomes of *T. cacao*.

| **Species** | **GenBank** | **Voucher** | **Accession** | **Country** |
| --- | --- | --- | --- | --- |
| ***Theobroma cacao*** | **OP354232** | **KUELAP-611** | **INDES06** | **Peru** |
| ***Theobroma cacao*** | **OP354233** | **KUELAP-619** | **INDES14** | **Peru** |
| ***Theobroma cacao*** | **MZ725364** | **KUELAP-638** | **INDES34** | **Peru** |
| ***Theobroma cacao*** | **OP354234** | **KUELAP-646** | **INDES50** | **Peru** |
| ***Theobroma cacao*** | **OP354235** | **KUELAP-655** | **INDES63** | **Peru** |
| ***Theobroma cacao*** | **MZ725365** | **KUELAP-659** | **INDES67** | **Peru** |
| ***Theobroma cacao*** | **OP354236** | **KUELAP-663** | **INDES71** | **Peru** |
| *Theobroma cacao* | JQ228387 | - | TARS 16664 | Trinidad and Tobago |
| *Theobroma cacao* | JQ228386 | - | TARS 12044 | Trinidad and Tobago |
| *Theobroma cacao* | JQ228381 | - | TARS 16664 | Trinidad and Tobago |
| *Theobroma cacao* | KY085907 | Wen12890 | - | - |
| *Theobroma cacao* | HQ244500 | - | - | Peru |
| *Theobroma cacao* | JQ228380 | - | TARS 16542 | Ghana |
| *Theobroma cacao* | JQ228389 | - | PI 275669 | - |
| *Theobroma cacao* | JQ228383 | - | TARS 16658 | Trinidad and Tobago |
| *Theobroma cacao* | JQ228382 | - | MIA 29885 | Peru |
| *Theobroma cacao* | JQ228385 | - | MIA 27956 | Suriname |
| *Theobroma cacao* | JQ228379 | - | Criollo-22 | Trinidad and Tobago |
| *Theobroma cacao* | HQ336404 | - | - | - |
| *Theobroma grandiflorum* | JQ228388 | - | 04-0254 | Puerto Rico |
| *Urena procumbens* | NC_054171 | - |  | China |
| *Grewia chungii* | NC_054166 | - | - | China |
| *Colona floribunda* | NC_054164 | - | - | China |
| *Abelmoschus esculentus* | KY635876 | - | - | United States |
| *Hibiscus taiwanensis* | NC_054167 | - | - | China |
| *Gossypium areysianum* | JN019795 | - | - | China |
| *Althaea officinalis* | KY085914 | - | - | - |
| *Bombax ceiba* | NC_037494 | - | - | China |
| *Excentrodendron hsienmu* | NC_054163 | - | - | China |
| *Pterospermum truncatolobatum* | NC_054168 | - | - | China |
| *Tilia amurensis* | MH169579 | - | - | South Korea |
| *Sterculia monosperma* | MN533974 | - | - | China |
| *Heritiera angustata* | NC_037784 | - | - | China |
| *Reevesia thyrsoidea* | NC_041441 | - | - | - |
| *Durio zibethinus* | MG138151 | - | - | China |
| *Mangifera indica* | KX871231 | PDBK_2014-0249 |  | - |
| *Tapiscia sinensis* | MF926267 | - |  | China |
| *Carica papaya* | EU431223 | - | - | - |
